# Surgery for mitral annular caseous calcification-related calcified amorphous tumor: a case report

**DOI:** 10.1186/s44215-023-00042-5

**Published:** 2023-05-15

**Authors:** Akimasa Morisaki, Yosuke Takahashi, Yoshito Sakon, Yosuke Sumii, Toshihiko Shibata

**Affiliations:** Department of Cardiovascular Surgery, Osaka Metropolitan University Graduate School of Medicine, Osaka, Japan

**Keywords:** Cardiac calcified amorphous tumor, Caseous calcification, Mitral valve annular calcification, Mitral valve surgery

## Abstract

**Background:**

A calcified amorphous tumor (a non-neoplastic tumor) with caseous calcification of the mitral annulus is a rare pathology that causes severe embolic events. We present a rare case of mitral valve surgery for a mitral annular caseous calcification-related calcified amorphous tumor found in cerebral infarction.

**Case presentation:**

A 69-year-old man was diagnosed with a mitral valve calcified amorphous tumor with mitral annular caseous calcification found in cerebral infarction. He was admitted because of acute multiple embolic cerebral infarctions. A search for the embolic source through transesophageal echocardiography revealed a mitral valve tumor raised from the posterior mitral valve leaflet on the side of the left ventricle. Computed tomographic cardiac angiography revealed a calcified mitral valve tumor invading the posterior mitral valve annulus and left ventricular muscle. Intraoperative findings revealed a mitral annular calcification-related calcified amorphous tumor with caseous calcification of the posterior leaflet and annulus, which was suspected. Therefore, we performed radical debridement of the mitral annular calcification and bioprosthetic mitral valve replacement with patch repair of the posterior mitral valve annulus 2 weeks after the onset of cerebral infarction. The patient recovered well post operation, without any embolic events.

**Conclusions:**

A calcified amorphous tumor with caseous mitral annulus calcification may be highly associated with embolic events. In this case, mitral valve replacement with annular patch repair may be a favorable procedure for preventing embolic events.

## Background

A calcified amorphous tumor (CAT) is a rare non-neoplastic tumor that commonly requires surgical intervention owing to embolic events [1]. Moreover, caseous mitral annular calcification (MAC) is a rare pathology that causes serious embolic events and is sometimes associated with CAT [2–4]. Here, we report a rare case of a patient who underwent bioprosthetic mitral valve replacement with patch annular repair for MAC-related CAT with caseous calcification found in cerebral infarction.

## Case presentation

A 69-year-old man, with combined pulmonary fibrosis, emphysema, and diabetes mellitus, who was being treated with insulin therapy and oral medications, suddenly developed dysarthria. He was diagnosed with multiple embolic cerebral infarctions using magnetic resonance imaging, and his symptoms were ameliorated by antiplatelet therapy after hospitalization. Magnetic resonance angiography showed no stenosis or occlusion without plaques in the cerebral and cervical arteries. Electrocardiography revealed no arrhythmia during hospitalization. However, transthoracic echocardiography revealed abnormal findings in the mitral valve posterior leaflet with tumor formation, restriction, and thickening of the middle posterior leaflet, as well as degenerative changes with the calcification of the mitral valve leaflets.

**Fig. 1:**
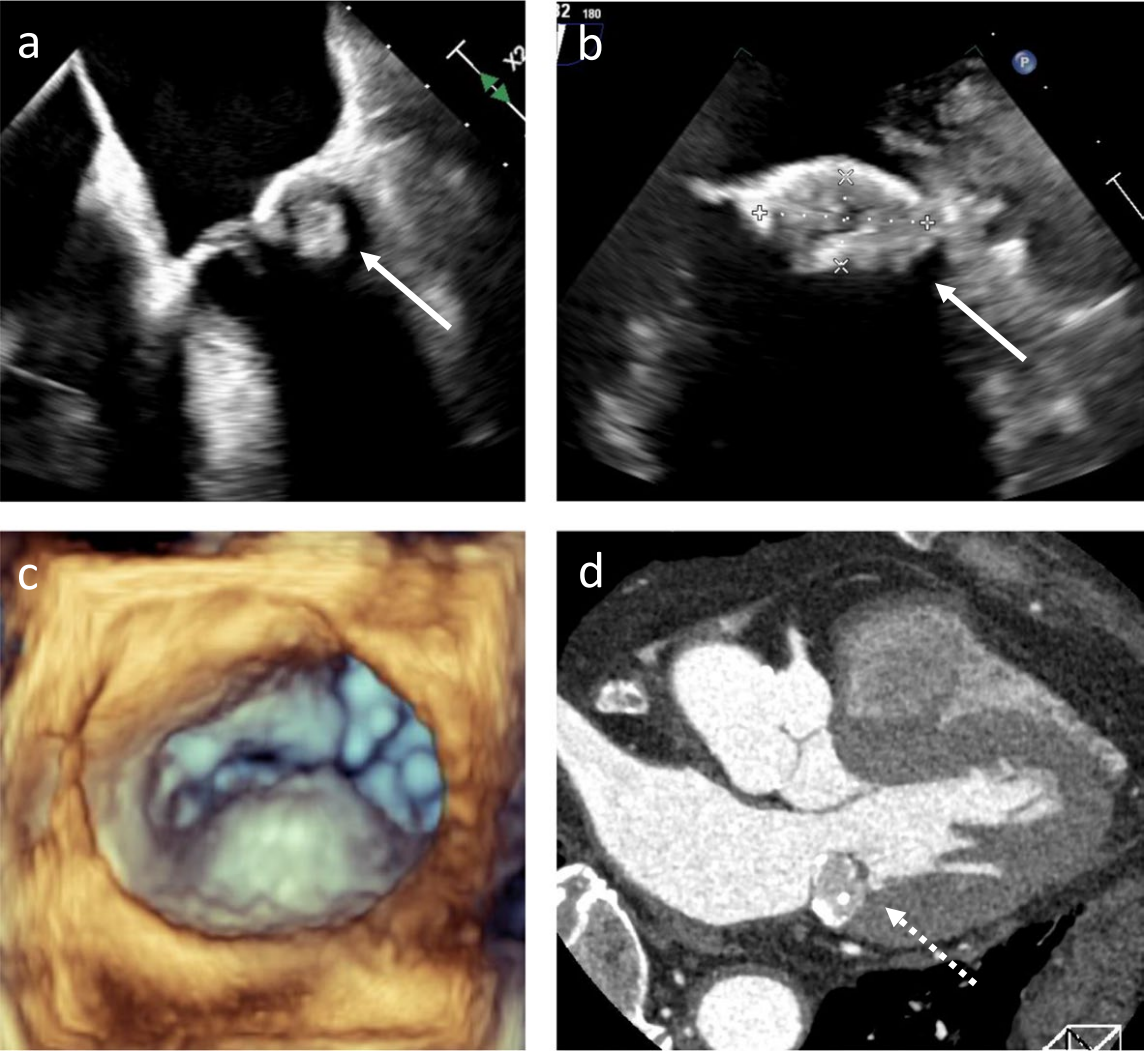
Preoperative examinational findings. **a**, **b** Transesophageal echocardiography. The white arrow indicates a posterior leaflet tumor that rose from the left ventricular side with a 12 × 22-mm diameter. **c** 3-dimensional transesophageal echocardiography shows a bulging of the middle posterior leaflet. **d** Cardiac computed tomography angiography. A white broken arrow indicates a calcified amorphous tumor that invaded the mitral valve annulus and left ventricular muscle

Additionally, transesophageal echocardiography revealed a mitral valve tumor with a 22-mm diameter that consisted of an irregular surface with internal heterogeneity brightness, raised from the middle posterior leaflet side of the left ventricle without mobility. No significant stenosis or regurgitation of the mitral valve was observed (Fig. 1a–c). However, cardiac computed tomography angiography revealed a calcified tumor with internal fluid formation invading the mitral valve annulus and left ventricular muscle, which was suspected to be MAC-related CAT (Fig. 1d). Hence, we considered surgery for MAC-related CAT because of the high possibility of recurrent embolic events over 2 weeks from the onset of cerebral infarction.

**Fig. 2:**
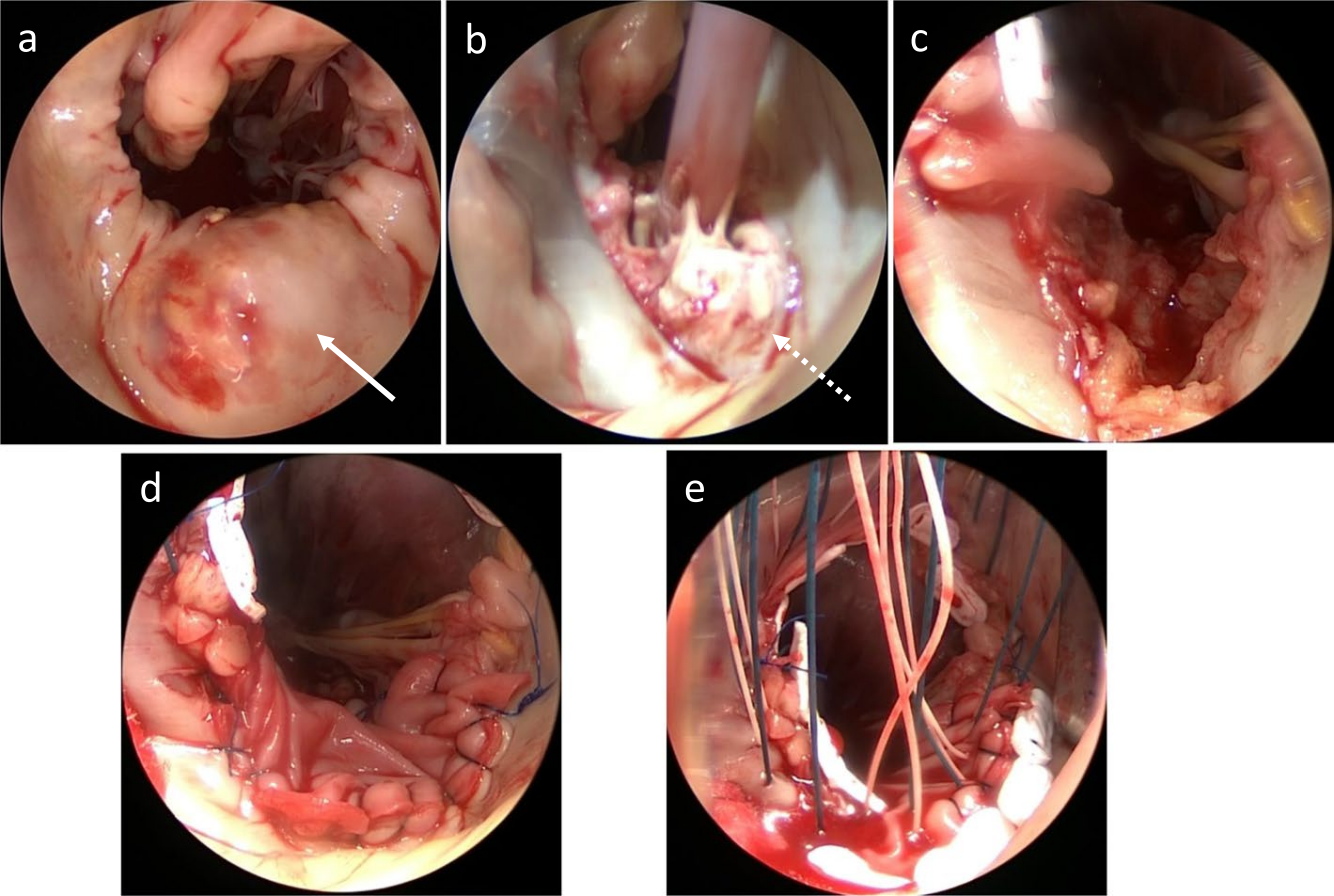
Intraoperative images. **a** Calcified amorphous tumor of the middle posterior leaflet with mitral annular calcification (white arrow). **b** Caseous calcification of mitral posterior leaflet and annulus (white broken arrow). **c** Debrided mitral annulus after removal and debridement of calcified amorphous tumor and mitral annular caseous calcification. **d** A patch repair with bovine pericardium of posterior mitral valve annulus. **e** A mixed suture technique consisted of non-everting mattress sutures in the middle posterior annulus and everting mattress sutures in the anterior annulus, and lateral and medial posterior annulus

**Fig. 3:**
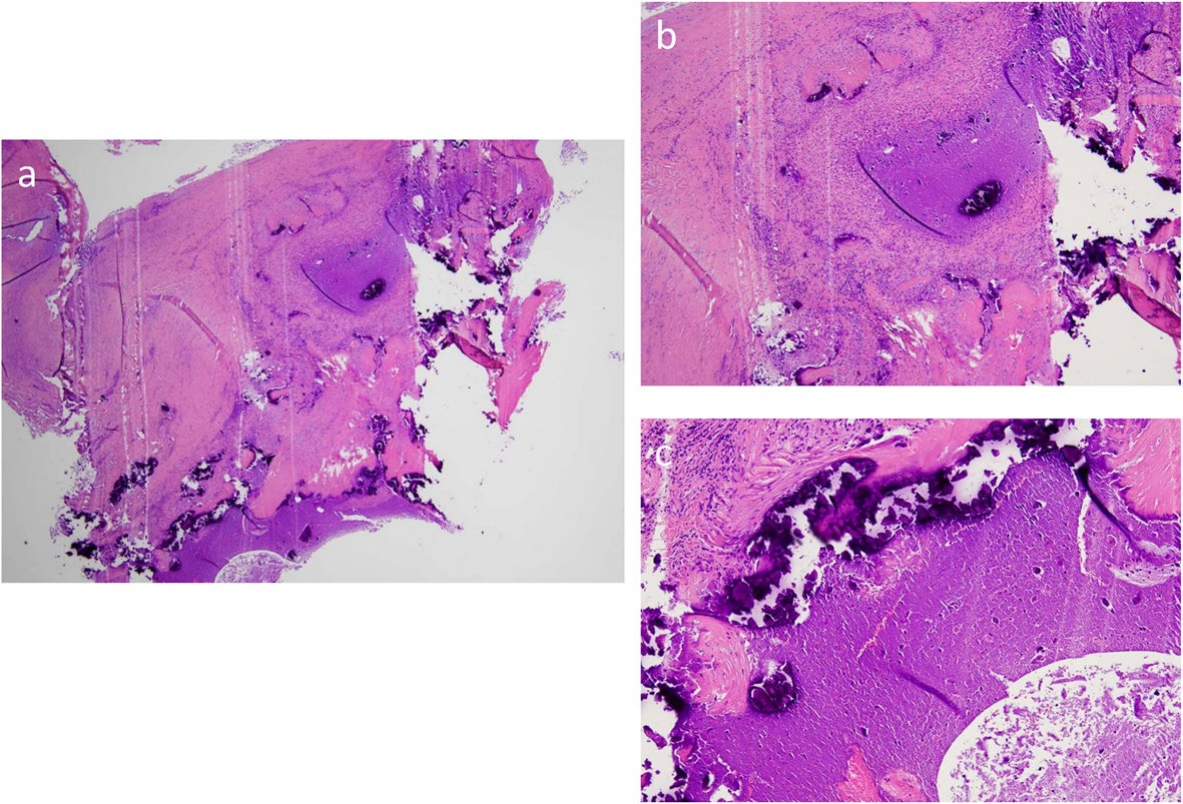
Histopathological findings reveal severe calcification and inflammatory infiltration with lymphocyte and plasmacyte of resected tumor of the mitral posterior leaflet. There were necrotic tissues without pathogens and neoplasm. These findings are consistent with those of a calcified amorphous tumor. **a** original magnification ×40, **b** ×200, **c** ×200. Hematoxylin and eosin staining

Under general anesthesia, the patient underwent cardiac tumor and MAC removal, mitral annulus patch repair with a bovine pericardial patch, and mitral valve replacement with a 29-mm Mosaic® (Medtronic, Minneapolis, MN, USA) through a median sternotomy with a conventional cardiopulmonary bypass. Mitral valve replacement was performed using a half-and-half technique consisting of a non-everting mattress and everting mattress sutures with spaghetti pledgets [5]. Intraoperative findings revealed a CAT in the middle posterior leaflet and MAC with caseous calcification, which was easily broken and removed (Fig. 2). The histopathological examination disclosed sterile inflammatory changes in the excised posterior leaflet, with necrosis and calcification, which consisted of CAT and caseous calcification (Fig. 3). Postoperative transthoracic echocardiography showed good mitral prosthetic valve function without perivalvular leakage or aneurysmal formation of the left ventricle. The patient was discharged uneventfully on postoperative day 10 and had been doing well without any embolic events for over half a year after the operation.

## Discussion and conclusions

CAT is an extremely rare non-neoplastic intracardiac tumor composed of calcified nodules and amorphous materials and is most commonly found in the mitral valve [1]. A recent study reported that CAT is usually associated with heart valve disease, hemodialysis, MAC, and diabetes mellitus [4]. In particular, patients with an end-stage renal disease requiring hemodialysis frequently develop CAT [1, 6]. In our case, a patient with diabetes mellitus who was receiving insulin treatment developed MAC-related CAT of the posterior mitral valve. Many previous reports indicated that CAT leads to embolic events, especially stroke, which may be caused by detachment of the mass, fibrin cap, or thrombus attached to the mass [1, 7, 8]. Nishiguchi et al. reported that the length of the CAT with cerebral infarction was significantly shorter than that of the CAT without cerebral infarction, suggesting fibrin cap embolization [1]. Additionally, MAC with caseous calcification is associated with a high cerebral infarction rate of over 19% compared with MAC without caseous calcification [2, 3]. We report a case of MAC-related CAT with caseous calcification, which caused high embolic events in cerebral infarction.

Correct diagnosis of CAT and MAC-related CAT may be difficult because the configuration of CAT sometimes resembles cysts, thrombi, tumors, or abscesses. Kanemitsu et al. recommended to use multimodality for effectively diagnosing CAT [6]. Echocardiography detected a mass in the mitral valve with central echolucencies surrounded by a hyperechogenic structure resembling liquefaction and exhibiting an irregular surface on the posterior mitral annulus. However, computed tomography revealed a well-defined hyperdense mass as a MAC-related CAT. In our case, transesophageal echocardiography showed only a mitral posterior leaflet tumor with a central liquid formation that suggested another cardiac tumor, such as myxoma or papillary fibroelastoma. However, computed cardiac angiography indicated the formation of a calcified tumor extending to the posterior mitral valve annulus and left ventricular muscle, suggesting a MAC-related CAT. Therefore, a multidisciplinary approach using various modalities is important for accurately detecting and diagnosing CAT or MAC-related CAT.

Radical surgical procedures for MAC-related CAT may be required to prevent recurrent embolic events due to residual calcifications. CAT can be treated with simple mitral valve replacement or repair if it arises from only mitral valve leaflets. However, some reports indicate the use of medication therapy with antiplatelet or warfarin to treat CAT [1, 8]. Additionally, surgery for severe, extensive MAC is challenging owing to technical issues such as non-penetrating calcium by direct sutures, perivalvular leakage, and atrioventricular dissociation [9, 10]. Some reports have shown that mitral valve surgery with preserved MAC or less debridement of the MAC may be useful for patients with severe MAC [11, 12]. However, MAC-related CAT is strongly associated with embolic stroke by a progression of calcification. This suggests a need to perform radical debridement of the embolic source, including MAC, to prevent recurrent embolic events [1]. Moreover, MAC with caseous calcification was associated with a higher cerebral infarction rate than MAC without caseous calcification [2, 3]. Previous reports recommend patch repair of the mitral valve annulus with radical debridement for MAC with caseous calcification to prevent recurrent embolic events [6, 13]. Therefore, we performed radical debridement and patch repair of the posterior mitral annulus for MAC-related CAT with caseous calcification that invaded the left ventricular muscle. During the follow-up, we did not observe any valve-related complications or thromboembolic events.

We present a rare case of MAC-related CAT with caseous calcification found in cerebral infarction. This case report demonstrates that CAT with caseous calcification may be strongly associated with embolic events. However, this patient had no embolic events during follow-up, following mitral valve replacement with annular patch repair for MAC-related CAT with caseous calcification.

## Data Availability

Not applicable.

## Abbreviations

CAT: Calcified amorphous tumor
MAC: Mitral annular calcification

## References

[1] Nishiguchi Y, Matsuyama H, Shindo A, Matsuura K, Niwa A, Hirota Y, et al. Cerebral embolism associated with calcified amorphous tumor: A review of cerebral infarction cases. Intern Med. 2021;60:2315–9. 33612675 10.2169/internalmedicine.6262-20 PMC8355388

[2] Dietl CA, Hawthorn CM, Raizada V. Risk of cerebral embolization with caseous calcification of the mitral annulus: Review Article. Open Cardiovasc Med J. 2016;10:221–32. 27990181 10.2174/1874192401610010221 PMC5120388

[3] Kim D, Shim CY, Hong GR, Jeong H, Ha J-W. Morphological and functional characteristics of mitral annular calcification and their relationship to stroke. PLoS One. 2020;15:e0227753. 31929595 10.1371/journal.pone.0227753 PMC6957171

[4] de Hemptinne Q, de Cannière D, Vandenbossche JL, Unger P. Cardiac calcified amorphous tumor: A systematic review of the literature. Int J Cardiol Heart Vasc. 2015;7:1–5. 28785635 10.1016/j.ijcha.2015.01.012 PMC5497183

[5] Takahashi Y, Sasaki Y, Hattori K, Kato Y, Motoki M, Morisaki A, et al. Successful surgical treatment for total circumferential aortic and mitral annulus calcification: application of half-and-half technique. Gen Thorac Cardiovasc Surg. 2016;64:418–21. 25385543 10.1007/s11748-014-0495-6

[6] Kanemitsu S, Bessho S, Sakamoto S, Yamamoto N, Ito H, Shimpo H. Calcified amorphous tumor with caseous calcification of mitral annulus in hemodialysis patients. Gen Thorac Cardiovasc Surg. 2020;68:1513–6. 32314150 10.1007/s11748-020-01363-w

[7] Greaney L, Chaubey S, Pomplun S, St Joseph E, Monaghan M, Wendler O. Calcified amorphous tumour of the heart: presentation of a rare case operated using minimal access cardiac surgery. BMJ Case Rep. 2011;2011:bcr0220113882. 22693315 10.1136/bcr.02.2011.3882 PMC3109694

[8] Nagao Y, Nakajima M, Hirahara T, Wada K, Terasaki T, Nagamine M, et al. Calcified Cerebral Embolism Due to a Calcified Amorphous Tumor. J Stroke Cerebrovasc Dis. 2018;27:e115–6. 29478938 10.1016/j.jstrokecerebrovasdis.2018.01.025

[9] Bedeir K, Kaneko T, Aranki S. Current and evolving strategies in the management of severe mitral annular calcification. J Thorac Cardiovasc Surg. 2019;157:555–66. 30385026 10.1016/j.jtcvs.2018.05.099

[10] Okada Y. Surgical management of mitral annular calcification. Gen Thorac Cardiovasc Surg. 2013;61:619–25. 23404309 10.1007/s11748-013-0207-7

[11] Di Stefano S, López J, Flórez S, Rey F, Arevalo A, San RA. Building a new annulus: a technique for mitral valve replacement in heavily calcified annulus. Ann Thorac Surg. 2009;87:1625–7. 19379934 10.1016/j.athoracsur.2008.09.014

[12] Salhiyyah K, Kattach H, Ashoub A, Patrick D, Miskolczi S, Tsang G, et al. Mitral valve replacement in severely calcified mitral valve annulus: a 10-year experience. Eur J Cardiothorac Surg. 2017;52:440–4. 28407126 10.1093/ejcts/ezx086

[13] Fong LS, McLaughlin AJ, Okiwelu NL, Nordstrand IAJ, Newman M, Passage J, et al. Surgical management of caseous calcification of the mitral annulus. Ann Thorac Surg. 2017;104:e291–3. 28838533 10.1016/j.athoracsur.2017.04.039

